# Draft Genome Sequence of Saccharomyces cerevisiae Strain P-684, Isolated from Prunus verecunda

**DOI:** 10.1128/MRA.00926-20

**Published:** 2020-10-08

**Authors:** Hiro Takahashi, Takayuki Yoshizaki, Hisashi Kondo, Taichiro Motomura, Masataka Murase, Anna Takahashi, Shuichi Fukuyoshi, Chiyoko Machida, Shin Kanamasa, Hiromi Shimizu, Shin-ichi Iwaguchi

**Affiliations:** a Graduate School of Medical Sciences, Kanazawa University, Kanazawa, Ishikawa, Japan; b Graduate School of Horticulture, Chiba University, Matsudo, Chiba, Japan; c Fundamental Innovative Oncology Core Center, National Cancer Center, Tokyo, Japan; d Faculty of Life Science and Biotechnology, Fukuyama University, Fukuyama, Hiroshima, Japan; e Faculty of Information Technologies and Control, Belarusian State University of Informatics and Radio Electronics, Minsk, Belarus; f College of Bioscience and Biotechnology, Chubu University, Kasugai, Aichi, Japan; g Institute of Medical, Pharmaceutical and Health Sciences, Kanazawa University, Kanazawa, Ishikawa, Japan; h Graduate School of Bioscience and Biotechnology, Chubu University, Kasugai, Aichi, Japan; i Nara Prefecture Institute of Industrial Development, Nara, Nara, Japan; j Faculty of Science, Nara Women's University, Nara, Nara, Japan; Broad Institute

## Abstract

Saccharomyces cerevisiae strain P-684 is a yeast isolated from the flowers of Prunus verecunda ‘Antiqua,’ producing high quantities of malic and succinic acids in sake brewing. Here, we report the draft genome sequence of P-684, enlightening the mechanisms of biosynthesis of these organic acids by this strain.

## ANNOUNCEMENT

Sake is a traditional Japanese alcoholic beverage made from fermented rice. One of its characteristics is that it is composed of various organic acids, especially malic acid and succinic acid, which are mostly produced by yeast and whose acidity greatly affects the taste of the beverage (1). We isolated Saccharomyces cerevisiae strain P-684 from the flowers of Prunus verecunda ‘Antiqua’ (cherry blossom, naranoyaezakura) using an enriched culture made from koji extract and used this yeast for sake brewing (2). The products brewed using P-684 had higher malic and succinic acid contents than did the sake brewed using other yeast strains (2). Thus, in this study, to characterize the above features at a molecular level, we analyzed the genome sequence of the yeast strain P-684.

P-684 cells were cultured in yeast extract-peptone-dextrose (YEPD) medium at 25°C. Then, they were collected, and their genomic DNA was isolated using a GenTLE (from yeast) high-recovery kit (TaKaRa Bio, Inc.). Using the DNA library prep kit of Beijing Genomics Institute (BGI), DNA fragmentation (average fragment length, 300 bp) using a g-TUBE device (Covaris, Inc.), end repair, A tailing, adaptor ligation, PCR, and library purification were conducted to prepare the library, according to the manufacturers’ instructions. The sequencing of the DNBseq DNA library (paired-end 150-bp reads) generated 16,621,910 reads on the DNBSEQ-G400 platform (MGI Tech, Shenzhen, China). The adapter sequence removal and low-quality base trimming, followed by *de novo* assembly, were conducted using the “trim read” and “*de novo* assembly” modules on the CLC Genomics Workbench, version 20.0.4 (Qiagen, Valencia, CA, USA), using the default parameters. Then, reference-guided scaffolding of the draft genome sequence was conducted using RaGOO version 1.1 (3), using the default parameters, and the S. cerevisiae S288c genome sequence (GenBank accession number GCF_000146045.2) as a reference. Among the RaGOO-generated scaffolds, a concatenated, unlocalized scaffold was discarded because the scaffold consisted of short, highly fragmented contigs that had no homology to the reference sequence. The resulting genome assembly was 11,492,082 bp long and was divided into 17 scaffolds comprising 901 contigs. The *N*_50_ values (contig and scaffold), GC content, and genome coverage were 34,008 bp and 883,925 bp, 38.1%, and 207.7×, respectively. Of the 1,759 benchmarking universal single-copy ortholog (BUSCO) genes, 97.2% (including 5.6% of duplicated genes) were found in the assembly, as calculated using BUSCO version 3.1.0 (4) and by using the parameter “sp=saccharomyces_cerevisiae_S288C” and the “Saccharomyceta_odb9” data set. The prediction of the coding region of the scaffolds was conducted using AUGUSTUS version 3.3.3 (5) with the following parameters: “noInFrameStop=true,” “genemodel=complete,” and “species=saccharomyces_cerevisiae_S288C.” The estimated number of genes in the draft genome was 5,277. Gene annotation was also performed using Trinotate version 3.2.1 (6) with the default parameters.

This genomic information could provide insights into the genetic basis of the characteristics of this yeast in sake brewing.

### Data availability.

The draft genome sequence and gene annotation for strain P-684 have been deposited in GenBank/ENA/DDBJ under accession number BLZQ01000000 (BLZQ01000001 to BLZQ01000017). The SRA/DRA/ERA accession number is DRA010485.
